# Kawasaki disease in a girl with turner syndrome: a remarkable association

**DOI:** 10.1186/1824-7288-40-24

**Published:** 2014-02-28

**Authors:** Stefano Stagi, Stefania Losi, Francesco Chiarelli, Maurizio de Martino, Fernanda Falcini

**Affiliations:** 1 Department of Health Sciences, University of Florence, Anna Meyer Children’s University Hospital, Florence, Italy; 2 Department of Internal Medicine, Rheumatology Section, Transition clinic, University of Florence, Florence, Italy; 3 Department of Paediatrics, University of Chieti, Chieti, Italy

## Abstract

We describe a girl with Turner syndrome, a genetic disorder of the X chromosome in a phenotypic female at increased risk of autoimmune and immunological diseases, who developed Kawasaki disease at the age of four years.

Given the possible relationship between these two disorders, we recommend suspecting Kawasaki disease in patients with Turner syndrome who present with persistent fever of unknown origin and who are not responsive to antibiotic therapy. Attention should be given to this phenomenon, as patients with Turner syndrome are themselves at higher risk of cardiovascular defects. Further studies are needed to better clarify this issue.

## Introduction

Kawasaki disease (KD) is a febrile systemic vasculitis mainly affecting young children and complicated by coronary artery aneurysms in approximately 25% of untreated patients [1]. Immunological abnormalities have been widely described in the acute phase of the disease [1]. Extensive immunological changes may be out of the normal range of responses to either viral or bacterial antigens [2], as has also been hypothesised for the mechanism of several autoimmune diseases [3]. Although patients with KD rarely develop a second immunological disorder, a study in a large cohort of affected children reported a higher incidence of coeliac disease than in the general population, strengthening the possible link between KD and other autoimmune disorders [4]. Moreover, KD has been described in association with a variety of immunodeficiency diseases, including chronic granulomatous disease, Wiskott-Aldrich syndrome, and hyperimmunoglobulin (Ig) E syndrome, conditions that are in turn associated with certain autoimmune diseases [5].

Turner syndrome (TS) is a genetic disorder (1 in 2000–2500 live-born female infants) resulting from the absence of an X chromosome or the presence of an abnormal X chromosome in a phenotypic female [6]. TS is characterised by certain typical features, such as growth retardation; gonadal insufficiency with infertility; and skeletal, cardiovascular, and renal abnormalities. This syndrome is also associated with autoimmune diseases such as diabetes, coeliac disease, and rheumatologic and thyroid disorders [7].

In the literature, only one case of a boy with mosaic 45, XY/45, XO who developed KD has been reported [8]. We describe a girl with TS who developed KD, and we hypothesise a possible relationship between these two pathologies.

## Case report

A 4-year-and-1-month-old Caucasian girl was admitted to our department with a 7-day history of persistent high fever, reaching 39°C despite antibiotic therapy, along with pharynx redness and a maculopapular rash on her trunk. Her past medical history revealed that she was born at the 33^rd^ week of a 2^nd^ dizygotic pregnancy.

At 3 months of age, she was operated on for intestinal occlusion, and during the first year of life, she underwent two surgical interventions for a diaphragmatic hernia. Her neurological and psychological development was slightly delayed.

At admission, she was extremely irritable, miserable, and pale. Her heart rate was 120/min; respiratory rate, 34/min; and brachial blood pressure, 80/50 mmHg. Her weight was 11.800 kg (< 3^rd^ centile), and her height was 93.6 cm (3^rd^ centile).

Extensive laboratory tests revealed an erythrocyte sedimentation rate (ESR) of 61 mm/h (nv < 15), a C-reactive protein (CRP) level of 9.71 mg/dL (nv < 0.5), a haemoglobin level of 12.3 g/dL, a white blood cell count of 11.90 × 10^9^ cells/L, a fibrinogen level of 573 mg/dL, a sodium level of 142 mEq/L, an ALT level of 164 IU/mL (nv = 10–40), and a gamma GT level of 78 IU/ml (nv < 40 IU/mL). Microbiological evaluation for bacterial and viral infections, including adenovirus, cytomegalovirus, parvovirus, herpes, and Epstein-Barr virus infections; Staphylococcus infection; and group A Streptococcus infection, yielded negative results. Throat and nasopharyngeal swabs for adenovirus culture were negative. Chest x-ray and abdominal ultrasound were unremarkable. Electrocardiography was normal, and 2D echocardiography showed mildly dilated coronary arteries associated with a bicuspid aortic valve, along with a moderate ascending aortic coarctation.

**Figure 1:**
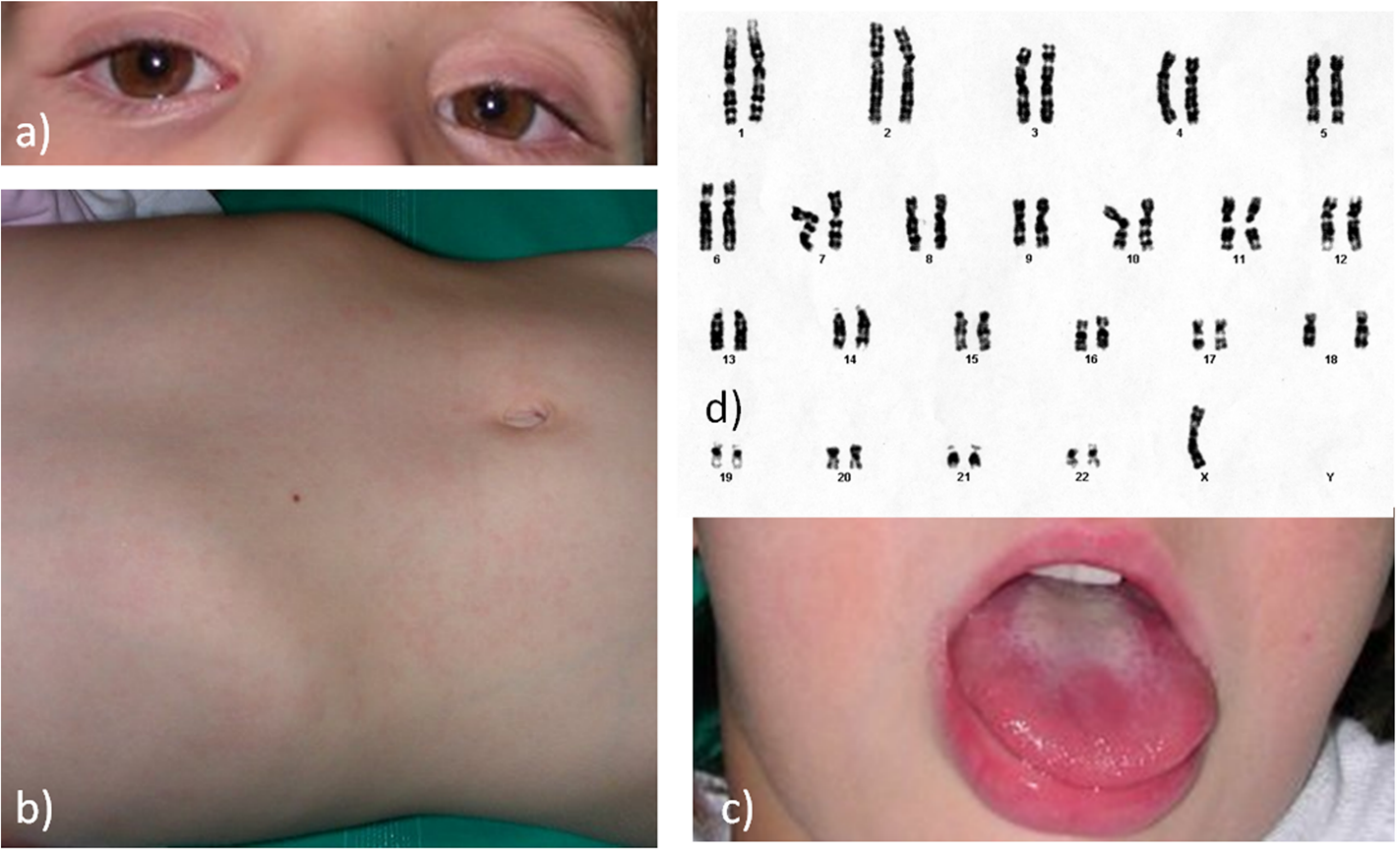
**Main characteristics of the patient during Kawasaki disease. a)** Bilateral, non-exudative conjunctivitis; **b)** diffuse erythematous maculopapular rash; **c)** mucositis and glossitis; **d)** 45, X karyotype.

On day 2 after admission, the girl developed non-exudative conjunctivitis, cervical lymphadenopathy, and mucositis, prompting us to diagnose KD (Figure 1a, b, and c). After intravenous immunoglobulin (IVIG; 2 g/Kg) and aspirin (50 mg/Kg in three divided doses) administration, her fever promptly dropped, and in the 2^nd^ week, her platelet count reached 873 × 10^9^. On day 15 after the onset of fever, the typical peeling of her digits supported a diagnosis of KD.

In parallel, due to the presence of dysmorphisms (cubitus valgus, a short fourth metacarpal, hyperconvex nails, and a high-arched palate), neonatal and heart malformations, and frequent episodes of serious otitis media, a genetic syndrome was assumed. Karyotype analysis revealed that the patient had a 45, XO karyotype, confirming the diagnosis of TS (Figure 1d).

A second step of laboratory tests was performed to exclude the presence of autoimmune diseases. Anti-gliadin (AGA) IgA and IgG, anti-endomysium (EmA) IgA, anti-transglutaminase (tTG) IgA, perinuclear antineutrophil cytoplasmic antibody (p-ANCA), anti-*Saccharomyces cerevisiae* antibodies (ASCAs), anticardiolipin (aCL) antibodies (ACAs), anti-thyroperoxidase (TPO), and antithyroglobulin (Tg) antibodies were negative. Antinuclear antibodies (ANAs) were positive, with a titre of 1:160.

During follow-up, at 5 years and 1 month old, the child developed transient low positivity for EmA and tTG, whereas at 8 years and 3 months old, she developed autoimmune thyroiditis, with negativity for AGA, EmA, tTG, p-ANCA, ASCAs, and ACAs.

## Discussion

TS is a disorder characterised by an increased risk of several autoimmune diseases, such as thyroid diseases, coeliac disease, inflammatory bowel disease, and rheumatologic and neurological disorders [6]. The mechanisms of this susceptibility are unknown. Non-random X chromosome inactivation has been hypothesised as being involved in the development of autoimmunity, and X chromosome monosomy has also been proposed as a common etiologic mechanism for certain autoimmune diseases [7,9].

In our patient, the association of TS and KD might be purely coincidental. However, another patient, with mosaic 45, XY/45, XO and who developed KD and mildly dilated coronary arteries, has been described in the literature [8]. Thus, we assume that TS might be potentially complicated by a higher risk of developing KD.

Furthermore, it is interesting to note that our patient developed transient positivity for EmA and tTG and subsequently developed autoimmune thyroiditis, a condition that is very common in TS. Moreover, our recent data have emphasised a possible link between KD and other autoimmune disorders, indicating a higher incidence of coeliac disease than in the general population [4] and strengthening past data on the concurrence of autoantibodies in both the acute and the convalescent phases of KD, such as aCL [10,11], ANCAs [10], ANAs, and antithyroid microsomal antibodies [12].

This association may also be of great concern because patients with TS (nearly 30%) may have and/or develop cardiovascular anomalies, such as aortic malformations and aneurysms, and particularly coronary artery disease (CAD), which is one of the most common causes of morbidity and mortality in TS and in KD [6]. Therefore, early cardiac imaging and an echocardiographic follow-up should be mandatory for TS patients [13].

CAD seems to be a common cardiac complication in both KD and TS, although the potential pathogenetic mechanisms are still unknown, and different sites of coronary arteries may be involved.

Several data also seem to suggest that long-term survivors of KD with or without coronary lesions have ongoing vascular inflammation and dysfunction and have a higher risk of accelerated atherosclerosis than do healthy subjects [14]. The production of cytokines, endothelin, and other vasoactive mediators, resulting in vascular endothelial changes, may have a permanent impact on vascular integrity, promoting the early onset of myocardial ischemia in adults who had KD in infancy [15,16]. Among the many pathophysiological factors that play an important role in the acceleration of atherosclerosis in vasculitis are enhanced oxidation processes, persistently activated T cells, and reduced numbers of T_reg_s [17]. In fact, a decrease in T_reg_s has been observed in KD [17] and in certain patients with TS [18].

Hence, KD could be another immunologic disorder that is potentially associated with TS. Because of the possible congenital and postnatal cardiovascular problems typical of TS, more attention must be given to patients with the syndrome who present with a prolonged fever of unknown origin that is refractory to broad-spectrum antibiotic treatment, along with high levels of inflammation.

## Consent

Written informed consent was obtained from the parents of the patient for publication of this case report and accompanying images. A copy of the written consent is available for review by the Editor-in-Chief of this journal.

## Competing interests

The authors declare that there are no conflicts of interest that could be perceived as prejudicing the impartiality of the research reported.

## Authors’ contributions

SS performed the endocrinological evaluation and wrote the paper. SL performed the gynaecological evaluation and participated in writing the paper. FC performed the endocrinological evaluation and participated in writing the paper. FF performed the rheumatologic evaluation. MdM participated in the rheumatologic evaluation and in writing the paper. All authors read and approved the final manuscript.
